# Retraction: FEZF1-AS1 is a key regulator of cell cycle, epithelial-mesenchymal transition and Wnt/β-catenin signaling in nasopharyngeal carcinoma cells

**DOI:** 10.1042/BSR-2018-0906_RET

**Published:** 2023-01-30

**Keywords:** cell cycle, EMT, FEZF1-AS1, nasopharyngeal carcinoma, Wnt/β-catenin signaling

This article is being retracted from *Bioscience Reports* at the request of the Editor-in-Chief and the Editorial Board following receipt of a notification from a reader, alerting the Editorial Board to multiple similarities between figures of this manuscript with those of other articles. The Figure 4B invasion/siFEZF1-AS1 panel and the Migration/NC panel show similarities with panels from Fig.3B and 5C from Li et al. 2019 (doi: 10.1042/BSR20181882), and the Figure 4A 0h/NC panel shows similarities with a panel from Fig. 7F of Li et al. 2021 (doi: 10.18632/aging.202847). The authors have been contacted with regards to the retraction and have not responded to the Journal's queries or the concerns raised. Given the extent of the issues raised, the Editorial Board stand by the decision to retract the article.

